# Discovery of Potential M2 Channel Inhibitors Based on the Amantadine Scaffold via Virtual Screening and Pharmacophore Modeling

**DOI:** 10.3390/molecules161210227

**Published:** 2011-12-08

**Authors:** Linh Tran, Sy Bing Choi, Belal O. Al-Najjar, Muhammad Yusuf, Habibah A. Wahab, Ly Le

**Affiliations:** 1 School of Biotechnology, Ho Chi Minh International University, Quarter 6, Linh Trung, Thu Duc District, Ho Chi Minh City 70000, Vietnam; Email: tran_linh_88@yahoo.com (L.T.); 2 Pharmaceutical Design and Simulation (PhDS) Laboratory, School of Pharmaceutical Sciences, Universiti Sains Malaysia, 11800 Minden, Pulau Pinang, Malaysia; Email: sybing@gmail.com (S.B.C.); belalnajjar@yahoo.com (B.O.A.-N.); myusuf_setiabudi@yahoo.com (M.Y.)

**Keywords:** M2 channel inhibitors, adamantane-based drugs, virtual screening, pharmacophore modeling

## Abstract

The M2 channel protein on the influenza A virus membrane has become the main target of the anti-flu drugs amantadine and rimantadine. The structure of the M2 channel proteins of the H3N2 (PDB code 2RLF) and 2009-H1N1 (Genbank accession number GQ385383) viruses may help researchers to solve the drug-resistant problem of these two adamantane-based drugs and develop more powerful new drugs against influenza A virus. In the present study, we searched for new M2 channel inhibitors through a combination of different computational methodologies, including virtual screening with docking and pharmacophore modeling. Virtual screening was performed to calculate the free energies of binding between receptor M2 channel proteins and 200 new designed ligands. After that, pharmacophore analysis was used to identify the important M2 protein-inhibitor interactions and common features of top binding compounds with M2 channel proteins. Finally, the two most potential compounds were determined as novel leads to inhibit M2 channel proteins in both H3N2 and 2009-H1N1 influenza A virus.

## 1. Introduction

**Figure 1:**
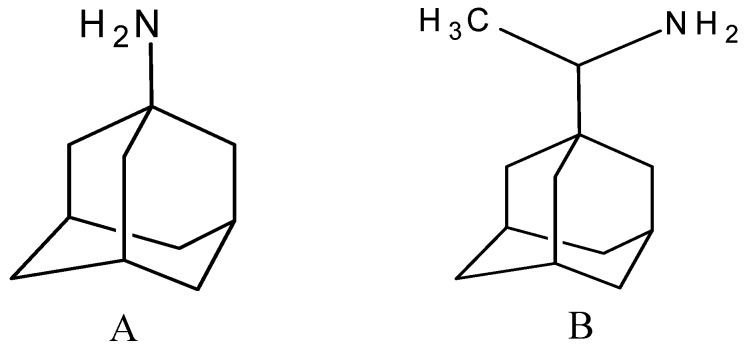
Structures of amantadine (**A**) and rimantadine (**B**).

A functional M2 channel protein is essential for the release of the flu virus’ genetic material inside an infected cell [1]. The M2 channel protein, which is a pH-sensitive proton channel, also plays a key role in virus replication and regulates virus morphology [2,3]. The two adamantane derivative-based drugs amantadine and rimantadine (Figure 1), which have been used as the first-choice antiviral drugs against community outbreaks, were the first antivirals licensed for effective against influenza A viruses [4]. However, since 2003, the frequency of amantadine resistance has increased significantly, from less than 5% to greater than 90% of isolated influenza A virus [5]. There is therefore a great urgent need to discover new types of M2 inhibitors for the development of new anti-inﬂuenza drugs due to the new mutations on M2 channel protein and the drug-resistance of amantadine and rimantadine [6]. The new drugs directed against M2 channel proteins should be more universal and effective than the previous ones.

Why have amantadine and rimantadine become resistant to influenza A virus recently? Site-directed mutagenesis and molecular dynamics simulations have been carried out to investigate the amantadine resistance in the trans-membrane domain of the M2 channel protein [7,8]. According to statistical data on resistant mutants, 70% to 80% of substitutions occur at position Ser31, and around 10% occur at positions Val27 and Ala30 *in vitro* and in clinical samples [7]. To solve the drug-resistance problem, a reliable molecular structure of M2 channel protein is a high priority for designing new drugs [10]. The M2 channel protein structures obtained experimentally in previous studies [11,12,13] have thus become the main targets for scientists and pharmacologists to find drugs against influenza A virus using structure-based drug design approaches [9,10]. One of them is the high-resolution nuclear magnetic resonance (NMR) spectroscopy structure by Schnell and Chou with the Protein Data Bank (PDB) code entry of 2RLF [14] that has successfully provided a full-length structure of H3N2 M2 channel protein. The 3D 2009-H1N1 M2 channel protein [15] built from sequence with the Genbank accession number of GQ385303 was also used in this current research.

In previous studies, drug binding affinities which modified the functional groups on amantadine did not reveal any details on how the ligands actually bind at the molecular level [16,17,18]. This research aims to search for drug candidates that are effective against the resistant strains of influenza A viruses and shed light on several important insight top hit M2 protein-inhibitor interactions. In this study, 200 drug candidates were designed by modifying or adding more functional groups to the amantadine scaffold and then used for virtual screening process [19]. After that, top 10 binding compounds were selected for further studied in pharmacophore analysis.

## 2. Results and Discussion

### 2.1. Binding Site Identification

Two possible binding sites for the M2 channel protein of influenza found in experimental studies are the drug binding locations [20]. The molecular docking results on both amantadine and rimantadine positioned inside and outside the M2 channel proteins partially supported the actual binding site location. The free energy of binding of amantadine and rimantadine inside the channel is generally lower than the binding outside the M2 channel proteins (*cf.* Table 1). 

**Table 1:** Summary of the lowest free energy of binding obtained in four cases of amantadine and rimantadine with H3N2 M2 channel protein and 2009-H1N1 M2 channel protein respectively.

Position	Ligand	Binding energy (kcal/mol)		Possible binding sites with M2 channel proteins	
		H3N2	2009-H1N1	H3N2	2009-H1N1
Inside	Amantadine	−6.85	−7.01	Hydrogen bond with Ala30	No Hydrogen bond
Inside	Rimantadine	−7.75	−7.57	Hydrogen bond with Ala30	No Hydrogen bond
				Hydrophobic interaction with Ile33	Hydrophobic interaction with Ile33
Outside	Amantadine	−5.03	−5.05	Hydrogen bond with Asp44	Hydrogen bond with Asp44
Outside	Rimantadine	−5.53	−4.92	Hydrogen bond with Asp44	Hydrogen bond with Asp44
					Hydrophobic interaction with Thr43

**Figure 2:**
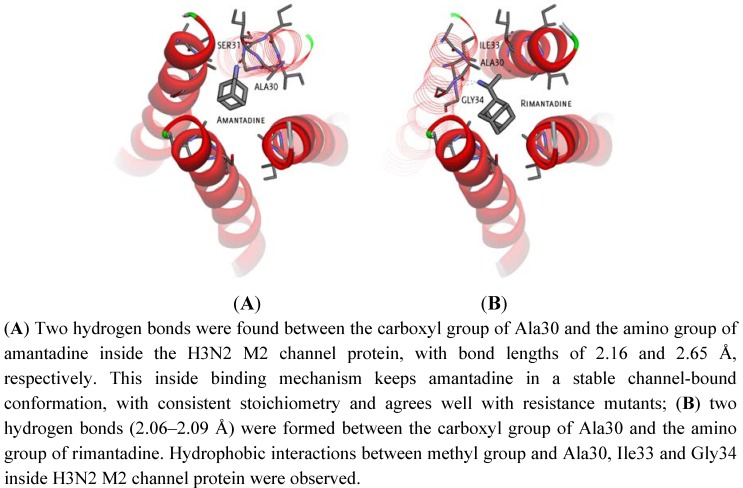
A close view of the interaction between amantadine and rimantadine inside H3N2 M2 channel protein respectively.

Comparing the two cases, the affinity of the inside channel binding is higher than that in the outside of the channel. The ligands binding inside the H3N2 M2 channel protein bind through a hydrogen bond with Ala30 (*cf.* Figure 2) but no formation of hydrogen bond with 2009-H1N1 M2 channel protein was observed for either amantadine or rimantadine. Meanwhile, the outside channel binding of ligands in both M2 channel proteins was largely unchanged in terms of the free binding energy and the location of the binding sites. This indicated that the binding between the ligands and the M2 protein structure is energetically more favourable and stable with the ligands (either amantadine or rimantadine) inside the channel than outside the M2 channel proteins.

Since the exact location of the functional adamantine binding site has been a source of controversy [20], for better design of novel M2 inhibitors and further comparison analysis, two binding positions were taken into consideration in this study.

### 2.2. Docking Results and Ranking Top Hit Compounds Binding Inside and Outside M2 Channel Proteins

The 200 compounds were divided into 7 groups based on the structure similarity. Their free energy of binding of 200 compounds to both H3N2 and 2009-H1N1 M2 channel proteins at two positions, namely inside and outside the M2 channel, is shown in the Table 2.

**Table 2:** Free energy of binding for 200 compounds.

Compound No.	2D Chemical Structure	INSIDE Free energy of binding (kcal/mol)		OUTSIDE Free energy of binding (kcal/mol)	
		H3N2	2009-H1N1	H3N2	2009-H1N1
**Group 1 (A1-A5) from [10]**					
A1	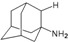	−6.85	−7.02	−5.03	−5.05
A2	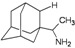	−7.75	−7.57	−5.53	−4.92
A3	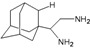	−8.48	−7.69	−5.50	−4.36
A4	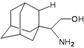	−8.09	−7.79	−5.25	−4.55
A5	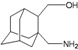	−8.16	−8.40	−4.85	−4.89
**Group 2 from [18]**					
B1	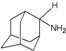	−6.85	−6.55	−5.53	−5.51
B2	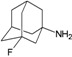	−7.10	−6.82	−5.81	−5.36
B3	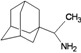	−7.82	−7.57	−5.17	−5.02
B4	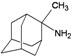	−7.44	−7.08	−5.65	−5.21
B5	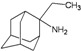	−7.70	−7.42	−5.53	−5.20
B6	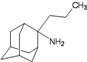	−7.97	−7.69	−5.52	−5.42
B7	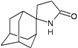	−8.32	−8.67	−6.15	−5.40
B8	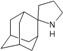	−8.53	−8.21	−5.78	−5.23
B9	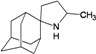	−8.58	−8.23	−6.02	−5.04
B10	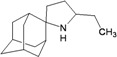	−8.93	−9.25	−5.81	−5.80
**Group 3 from [21]**					
1a	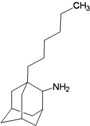	−8.70	−8.48	−6.04	−6.11
1b	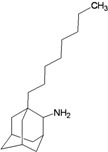	−9.46	−8.91	−6.49	−5.51
1c	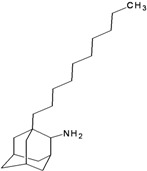	−9.39	−8.89	−6.55	−3.04
1d	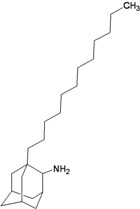	−9.20	−9.62	−4.71	−2.60
1e	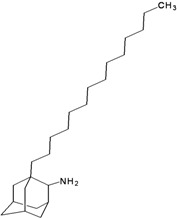	−8.99	−9.81	−4.45	−0.30
1f	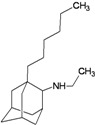	−8.73	−8.54	−5.45	−5.01
1g	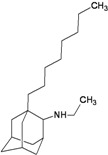	−9.91	−9.80	−5.32	−2.52
1h	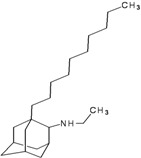	−9.53	−9.07	−4.62	−0.15
1i	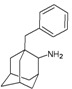	−10.29	−10.02	−7.33	−6.72
2a	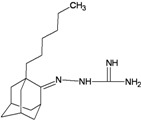	−10.47	−10.14	−5.79	−3.63
2b	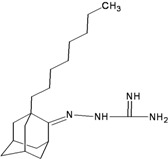	−10.48	−10.23	−5.42	−3.27
2c	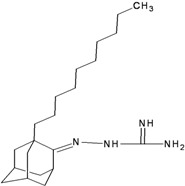	−10.69	−11.23	−3.47	+3.03
2d	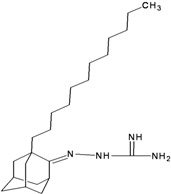	−10.26	−11.35	−4.61	−2.52
2e	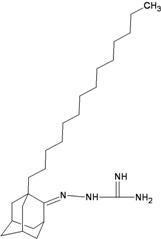	−9.97	−11.14	−0.34	−1.90
2f	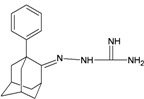	−10.30	−10.61	−5.98	−5.56
2g	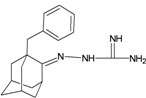	−11.21	−11.48	−6.16	−5.60
3a	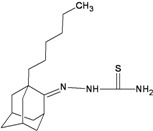	−10.18	−10.40	−4.96	−3.93
3b	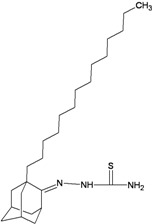	−9.17	−10.81	+3.01	+4.05
III	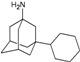	−9.96	−9.62	−6.72	−7.27
IV	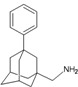	−10.20	−10.10	−6.57	−7.20
V	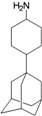	−9.71	−9.44	−5.88	−5.17
**Group 4 from [22,23]**					
M1	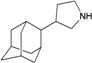	−8.55	−8.44	−5.77	−5.69
M2	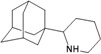	−9.03	−8.75	−5.53	−5.89
M3	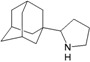	−8.58	−8.43	−5.61	−5.63
M4	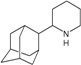	−9.05	−8.87	−5.85	−5.81
M5	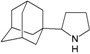	−8.60	−8.23	−5.56	−5.64
M6	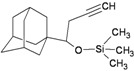	−8.47	−8.95	−3.62	−4.76
M7	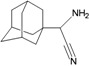	−8.21	−7.97	−5.53	−5.23
M8	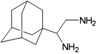	−8.65	−7.95	−5.97	−4.57
M9	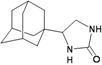	−9.14	−9.20	−4.91	−5.88
M10	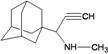	−8.47	−8.25	−5.05	−5.06
TaM11	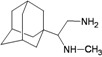	−8.44	−8.00	−5.62	−4.10
M12	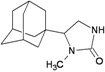	−9.38	−9.59	−5.03	−5.97
M13	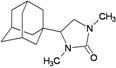	−9.42	−9.31	−4.16	−5.89
M14	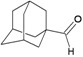	−6.73	−6.89	−5.07	−4.40
M15	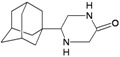	−9.09	−8.91	−5.75	−5.70
M16	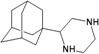	−9.04	−8.27	−5.73	−5.04
M17	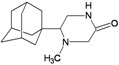	−9.58	−9.48	−4.19	−5.69
M18	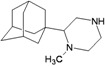	−9.70	−8.87	−4.34	−4.71
M19	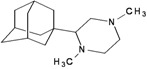	−10.03	−9.33	−4.23	−4.67
M20	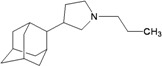	−9.48	−9.27	−5.94	−5.14
M21	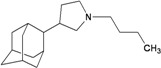	−9.72	−9.61	−5.91	−5.12
M22	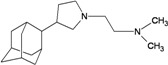	−10.19	−9.49	−6.15	−4.09
M23	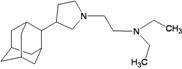	−10.63	−10.08	−5.49	−2.87
M24	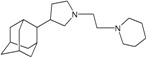	−11.59	−11.23	−7.26	−3.36
Bananin	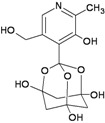	−9.67	−9.39	−3.83	−4.40
Iodobananin(IBN)	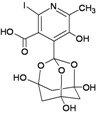	−9.60	−10.16	−4.96	−5.36
Eubananin(EUB)	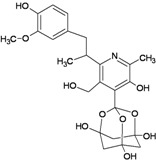	−8.54	−8.51	+1.71	+3.06
Vanillinbananin(VBN)	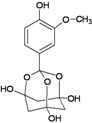	−8.70	−9.22	−4.58	−4.74
**Group 5 from [24]**					
D1	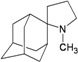	−8.48	−8.26	−5.38	−5.06
D2	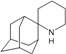	−7.83	−7.94	−5.58	−5.20
D3	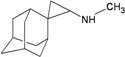	−7.38	−6.91	−4.27	−3.86
D4	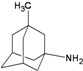	−7.46	−7.29	−5.69	−5.53
D5	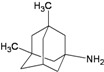	−8.01	−7.93	−5.61	−5.70
D6	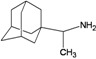	−7.79	−7.59	−5.17	−5.15
D7	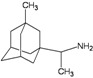	−8.54	−8.27	−5.72	−5.60
D8	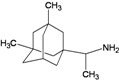	−9.10	−8.86	−6.12	−5.72
D9	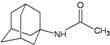	−7.12	−7.37	−5.51	−4.77
D10	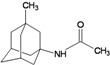	−7.75	−7.84	−4.30	−4.93
D11	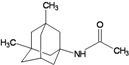	−8.17	−8.50	−4.78	−4.98
D12	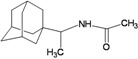	−7.96	−8.65	−4.47	−5.19
D13	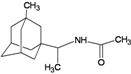	−8.77	−8.86	−4.69	−5.93
D14	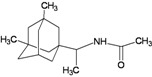	−9.28	−9.62	−5.05	−6.13
D15	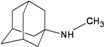	−6.99	−6.83	−4.97	−4.88
D16	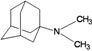	−7.17	−7.02	−5.01	−4.50
**Group 6 from [25,26]**					
N1	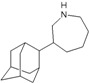	−9.38	−9.74	−5.51	−5.51
N2	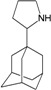	−8.57	−8.26	−5.65	−5.17
N3	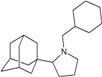	−12.09	−11.76	−6.72	−6.72
N4	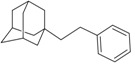	−9.62	−9.91	−5.91	−6.21
N5	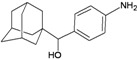	−9.76	−9.87	−5.42	−4.82
N6	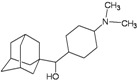	−10.44	−10.31	−5.55	−3.69
N7	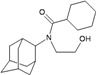	−10.15	−10.70	−5.54	−5.25
N8	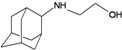	−7.37	−7.06	−5.22	−4.77
N9	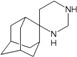	−8.50	−7.58	−5.50	−5.06
N10	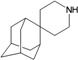	−8.83	−8.50	−5.18	−5.23
N11	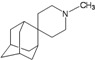	−9.24	−8.97	−5.25	−5.36
N12	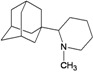	−9.53	−9.34	−5.94	−6.46
N13	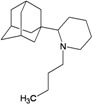	−10.49	−10.41	−5.76	−5.29
N14	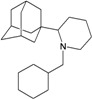	−12.25	−12.10	−6.68	−3.99
N15	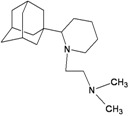	−10.98	−10.12	−5.68	−4.78
N16	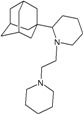	−12.50	−12.13	−6.44	−3.86
N17	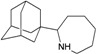	−9.73	−9.47	−4.49	−3.30
N18	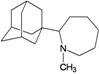	−9.97	−9.73	−3.64	−3.36
N19	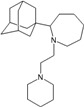	−12.52	−12.00	−1.18	+4.19
N20	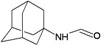	−6.98	−7.26	−5.08	−4.60
N21	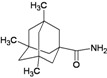	−9.06	−9.30	−5.20	−5.64
**Group 7**					
1	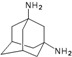	−7.50	−7.72	−5.16	−5.06
2	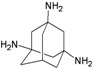	−8.21	−6.87	−6.24	−4.37
3	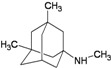	−8.08	−8.18	−5.35	−5.27
4	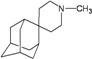	−9.24	−8.97	−5.25	−5.36
5	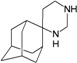	−8.41	−7.75	−5.21	−4.69
6	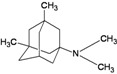	−8.46	−8.25	−5.20	−4.93
7	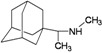	−7.62	−7.45	−5.00	−4.42
8	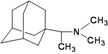	−8.14	−7.78	−5.09	−4.64
9	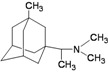	−8.85	−8.42	−5.27	−5.05
10	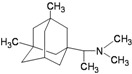	−9.35	−8.97	−5.59	−5.35
11	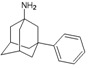	−9.23	−9.51	−6.17	−6.96
12	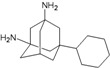	−10.59	−10.11	−7.13	−6.92
13	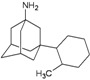	−10.51	−10.28	−6.73	−7.62
14	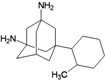	−11.21	−10.61	−7.13	−7.30
15	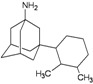	−10.67	−10.46	−6.49	−7.68
16	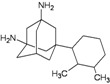	−11.22	−10.84	−7.25	−7.50
17	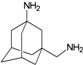	−8.04	−8.23	−5.36	−5.18
18	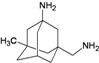	−8.80	−8.00	−6.37	−6.03
19	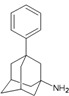	−9.16	−9.47	−6.17	−6.95
20	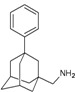	−9.67	−9.67	−6.12	−6.73
21	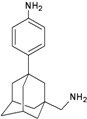	−10.00	−10.50	−5.99	−5.41
22	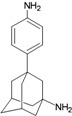	−9.64	−10.03	−5.55	−5.32
23	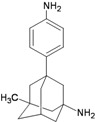	−10.02	−10.79	−5.77	−5.71
24	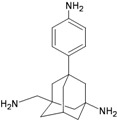	−10.52	−11.20	−5.88	−5.57
25	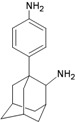	−9.30	−9.69	−5.52	−5.51
26	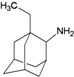	−7.80	−7.57	−5.57	−5.49
27	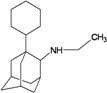	−9.95	−9.93	−6.15	−6.61
28	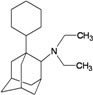	−10.81	−10.77	−5.66	−3.76
29	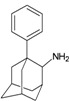	−8.95	−9.23	−5.31	−6.24
30	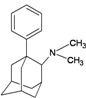	−9.43	−10.12	−5.68	−5.38
31	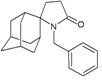	−10.47	−10.53	−6.06	−5.32
32	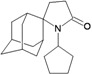	−10.60	−10.70	−6.11	−5.99
33	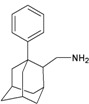	−9.51	−9.83	−4.80	−5.36
34	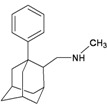	−9.70	−9.97	−5.37	−5.62
35	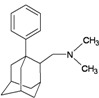	−10.00	−10.23	−5.69	−5.71
37	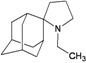	−8.78	−9.17	−5.53	−5.63
38	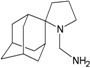	−9.45	−8.43	−5.86	−5.89
39	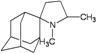	−9.18	−8.77	−5.32	−5.40
40	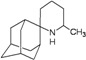	−8.53	−8.13	−5.58	−5.25
41	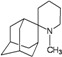	−9.10	−8.78	−5.55	−5.56
42	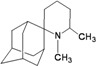	−9.61	−9.25	−5.71	−5.73
43	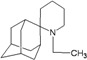	−9.42	−9.01	−5.26	−5.04
44	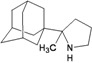	−9.07	−9.14	−5.01	−5.55
45	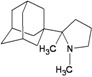	−9.51	−9.55	−3.97	−5.73
46	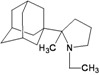	−9.63	−9.81	−3.46	−5.69
47	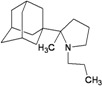	−9.90	−10.25	−3.53	−5.59
48	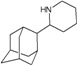	−9.01	−8.66	−5.96	−5.91
49	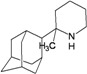	−9.25	−9.21	−5.46	−5.55
50	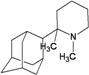	−9.85	−9.61	−5.15	−5.04
51	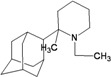	−10.11	−9.83	−4.14	−4.91
52	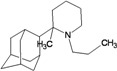	−10.41	−10.86	−4.17	−4.56
53	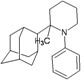	−11.35	−11.97	−5.26	−3.29
54	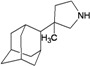	−9.15	−8.86	−5.69	−5.19
55	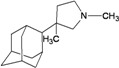	−9.55	−9.34	−5.37	−5.06
56	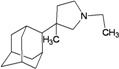	−9.60	−9.59	−5.39	−4.77
57	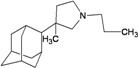	−9.85	−9.70	−5.45	−4.42
58	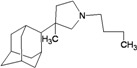	−10.10	−10.01	−5.00	−4.37
59	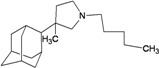	−10.74	−10.33	−4.91	−5.01
60	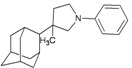	−10.76	−11.31	−5.47	−3.82
61	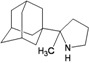	−9.14	−9.23	−4.59	−5.69
62	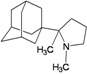	−9.50	−9.15	−4.04	−5.33
63	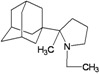	−9.88	−9.55	−3.69	−4.42
64	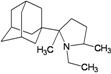	−10.15	−10.49	−3.49	−3.72
65	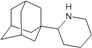	−9.03	−8.74	−5.55	−5.90
66	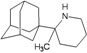	−9.46	−9.27	−4.12	−5.28
67	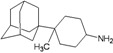	−10.06	−9.72	−4.47	−3.54
68	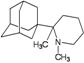	−9.92	−9.78	−4.13	−5.56
69	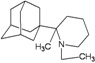	−10.31	−10.19	−3.75	−4.61
70	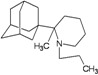	−10.62	−10.38	−3.57	−4.70
71	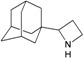	−8.30	−7.81	−5.66	−5.55
72	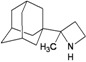	−8.60	−8.11	−4.77	−4.77
73	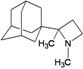	−8.84	−8.76	−4.35	−4.26
74	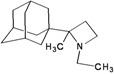	−9.25	−9.04	−3.86	−3.89
75	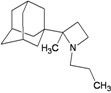	−9.62	−9.25	−4.39	−3.78
76	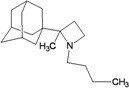	−9.80	−9.45	−4.07	−3.80
77	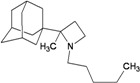	−10.10	−9.82	−3.95	−1.91
78	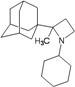	−10.90	−10.95	−4.60	−4.48
79	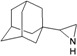	−7.44	−7.08	−5.08	−4.83
80	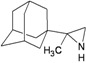	−7.87	−7.66	−4.93	−4.56
81	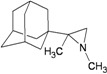	−7.98	−7.92	−4.38	−4.91
82	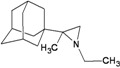	−8.29	−8.11	−4.34	−4.59
83	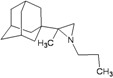	−8.76	−8.61	−4.56	−4.41
84	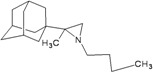	−9.01	−8.82	−4.65	−4.30
85	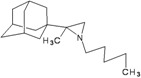	−9.35	−8.77	−4.66	−4.21
86	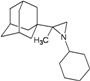	−10.19	−10.15	−5.37	−5.29
87	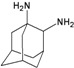	−7.91	−7.15	−6.62	−5.94
88	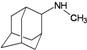	−6.94	−6.63	−5.34	−4.58
89	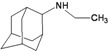	−7.23	−6.82	−5.37	−4.59
90	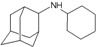	−9.23	−9.04	−6.38	−5.84
91	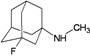	−7.16	−7.02	−5.26	−5.16
92	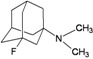	−7.40	−7.65	−5.69	−5.22
93	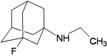	−7.41	−7.01	−5.45	−4.74
94	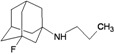	−7.67	−7.39	−5.57	−4.91
95	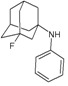	−8.86	−9.15	−5.65	−5.72
96	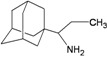	−8.00	−7.62	−5.23	−4.88
97	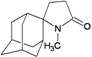	−8.66	−8.92	−5.16	−5.90
98	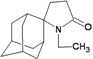	−8.90	−9.01	−5.06	−6.16
99	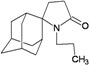	−9.37	−9.51	−5.20	−6.42
100	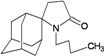	−9.74	−9.81	−5.39	−6.50

The selection of top binding compounds was mostly based on choosing the common compounds in both positions which have the lowest free energy of binding with the 2009-H1N1 M2 channel protein. In comparison with amantadine and rimantadine, the top 10 compounds were ranked based on the lowest free energy of binding obtained from docking which indicates a better binding affinity. Two groups were selected from the lowest energy of binding from virtual screening inside and outside the M2 channel proteins, respectively. Table 3 shows that the affinity of M2 drug candidates binding inside the channel is much higher than binding outside the M2 channel proteins. 

**Table 3:** Top 10 binding compounds inside and outside H3N2 M2 channel protein and 2009-H1N1 M2 channel protein according to free energy of binding (kcal/mol) from virtual screening.

Inside Ranking Comp.	Top 10 binding compounds (inside M2 channel protein)	Free energy of binding (kcal/mol)			Outside Ranking Comp.	Top 10 binding compounds(outside M2 channel protein)	Free energy of binding (kcal/mol)	
		H3N2	2009-H1N1				H3N2	2009-H1N1
I1	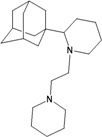	−12.50	−12.13		O1	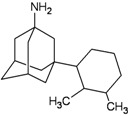	−6.49	−7.68
I2	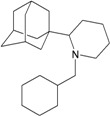	−12.25	−12.10		O2	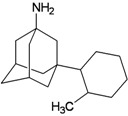	−6.73	−7.62
I3	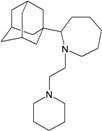	−12.52	−12.00		O3	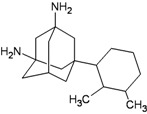	−7.25	−7.50
I4	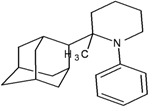	−11.35	−11.97		O4	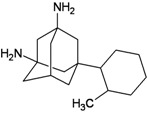	−7.13	−7.30
I5	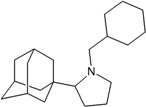	−12.09	−11.76		O5	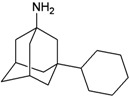	−6.72	−7.27
I6	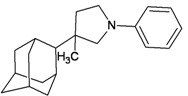	−10.76	−11.31		O6	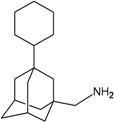	−6.57	−7.20
I7	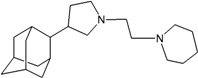	−11.59	−11.23		O7	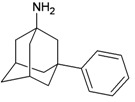	−6.17	−6.96
I8	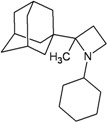	−10.90	−10.95		O8	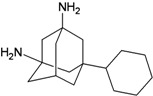	−7.13	−6.92
I9	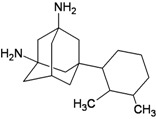	−11.22	−10.84		O9	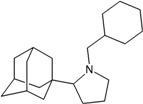	−6.72	−6.72
I10	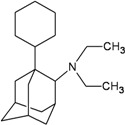	−10.81	−10.77		O10	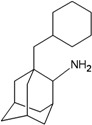	−7.33	−6.72

**Figure 3:**
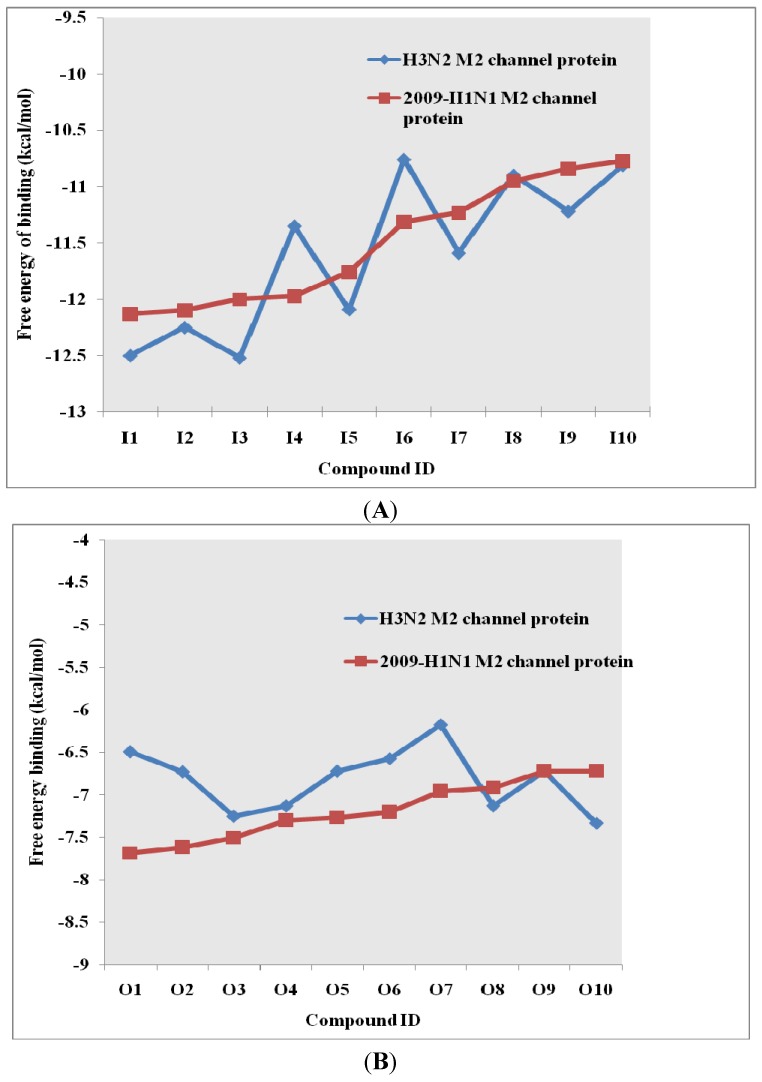
Free energy of binding of top 10 compounds: (**A**) inside M2 channel proteins; (**B**) outside M2 channel proteins.

The group inside the channel generally binds with high affinity through hydrophobic interactions inside the M2 channel proteins, speciﬁcally to hydrophobic residues Ile33, Val27, Ala30. In particular, for compound I9, a high possibility of forming hydrogen bonds was predicted for the amino group of I9 and two residues Gly34 or Ile34, whereas the methyl groups interact with the hydrophobic part of the M2 channel proteins (Ala30 and Ile33). Subsequently, I9, with high affinity, was selected for further pharmacophore analysis of the top hits’ M2 protein-inhibitor interactions. Besides, the group binding on the outside channel basically binds with lower affinity through hydrogen bonds with Asp44 in every compound in either of the M2 protein types, and some hydrophobic interactions with Arg45, Leu46, Phe47 and Phe48. Interestingly, both compounds I5 and I9 are identical to O9 and O3 respectively (*cf.* Table 2). Therefore, these two compounds were selected to be top hits for either the inside or outside binding M2 channel proteins for further pharmacophore analysis of specific drug target-protein interactions. Moreover, the free energy of binding of the top 10 compounds binding inside and outside the M2 channel proteins was compared in detail (*cf.* Figure 3).

### 2.3. Pharmacophore Analysis for Top hits M2 Channel-Inhibitor Interactions

LigandScout presents the interactions between M2 channel proteins and ligands, as well as with some excluded volume spheres corresponding to their 3D complex structures. In terms of potential drug-target binding in this study, the top two selected hit compounds were visualized clearly for the possible interactions with critical residues in the M2 channel proteins. The generated four pharmacophore models of two highly potent binding compounds both inside and outside the M2 channel proteins with their geometrical constraints and active sites were represented in Table 4.

**Table 4:** Structure-based hypotheses were superimposed on the active site of 3D structure of M2 channel proteins. Hydrogen Bond Acceptor (HBA) is shown as green vectored spheres, Hydrophobic (H) as light blue spheres (for interpretation of the references to colour in this ﬁgure legend, the reader is referred to the web version of this article.)

Cartoon Representation of M2 protein complex	Protein-Ligand Interactions	Hypothesis
***I5 inside M2 channel***		
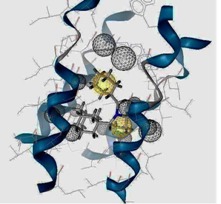	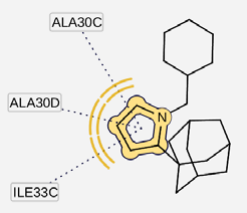	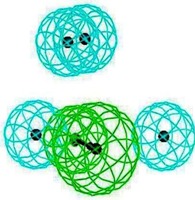
***I9 inside M2 channel***		
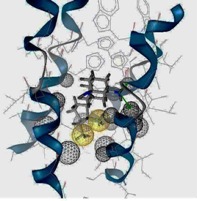	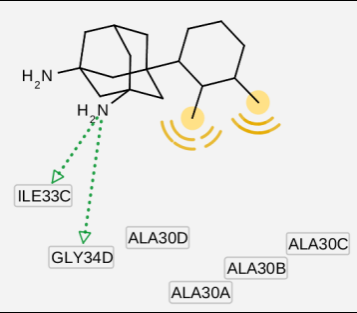	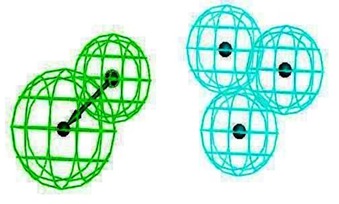
***I5 outside M2 channel***		
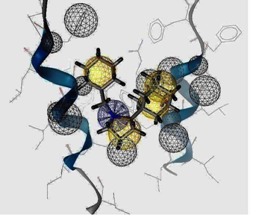	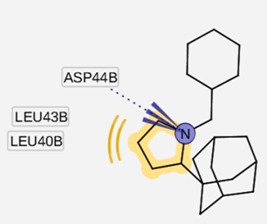	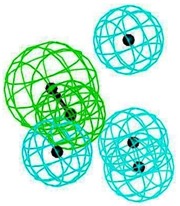
***I9 outside M2 channel***		
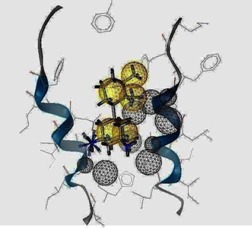	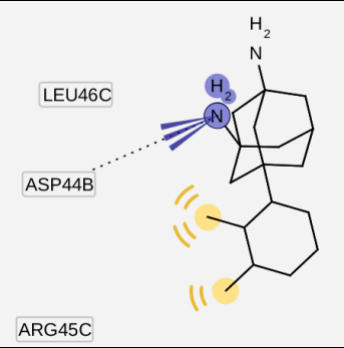	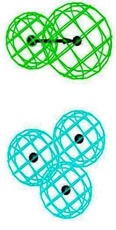

As shown in Table 4, for the I5 inside the M2 channel protein complex, the generated pharmacophore contains one hydrophobic (H) chemical feature which points towards Ala30 and Ile33. A four features hypothesis was generated from the I9 inside the M2 channel complex, which comprises one HBA towards Ile33 and Gly34, and three H groups mostly oriented towards Ala30 with 14 excluded volume spheres. A five features hypothesis was generated from the I5 outside the M2 channel complex, including one HBA toward Asp44 and four H groups pointed towards Leu40 and Leu43 with 11 excluded volume spheres. The I9 outside the M2 channel complex produced a four features hypothesis consisting of one HBA and three H groups with nine excluded volume spheres. The HBA group pointed towards the Asp44 while the H groups pointed towards Arg45 and Leu46, respectively. Comparing the above four pharmacophore models, the HBA and H groups from all the models were pointed towards Ala30, Ile33, Gly34 inside the M2 channel protein and Asp44, Leu40, Leu43, Arg 45 and Leu46 outside the M2 channel, which plays a major role in inhibiting M2 channel protein activity, respectively. The results suggested that these residues are extremely important binding sites for novel M2 channel inhibitors.

### 2.4. Common Pharmacophore Features of the Top Binding Compounds

**Figure 4:**
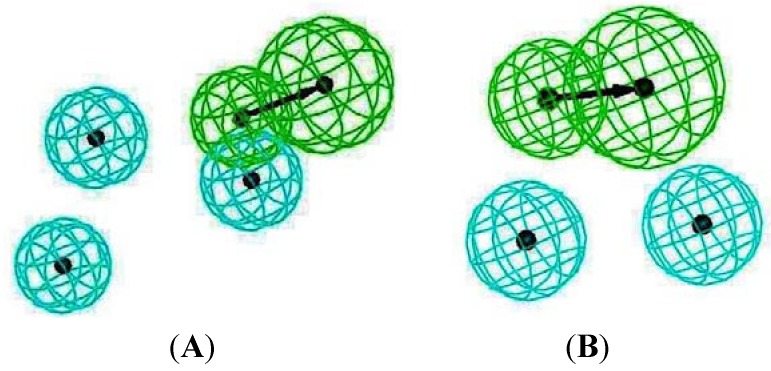
Common pharmacophore features of the top 10 inside binding (**A**) and outside binding (**B**) compounds. Hydrogen Bond Acceptor (HBA) is presented as green vectored spheres and Hydrophobic (H) as light blue spheres (for interpretation of the references to colour in this ﬁgure legend, the reader is referred to the web version of this article.)

The best hypothesis from Hip-Hop was chosen from seventeen hypotheses of the inside group and eighteen hypotheses from the outside group using Common Feature Pharmacophore Generation/Discovery Studio. As a result, the two representative pharmacophore features of the top 10 inside binding and outside binding compounds were identified (*cf.* Figure 4). For the group of compounds binding inside the M2 channel proteins, the generated pharmacophore contains one HBA and three H chemical features. Similarly, a three features hypothesis was generated from the group of outside binding compounds, which was composed of one HBA and two H groups. Hence, HBA and H features are considered as important chemical features to discover novel M2 channel inhibitors. Consistently with what predicted was by LigandScout, common features from Discovery Studio also indicate that all top binding compounds either inside or outside fit the common features having the same pharmacophore functional groups that can interact with critical residues in both the H3N2 and 2009-H1N1 M2 channel proteins.

## 3. Experimental

### 3.1. Proteins and Inhibitor Preparation

**Figure 5:**
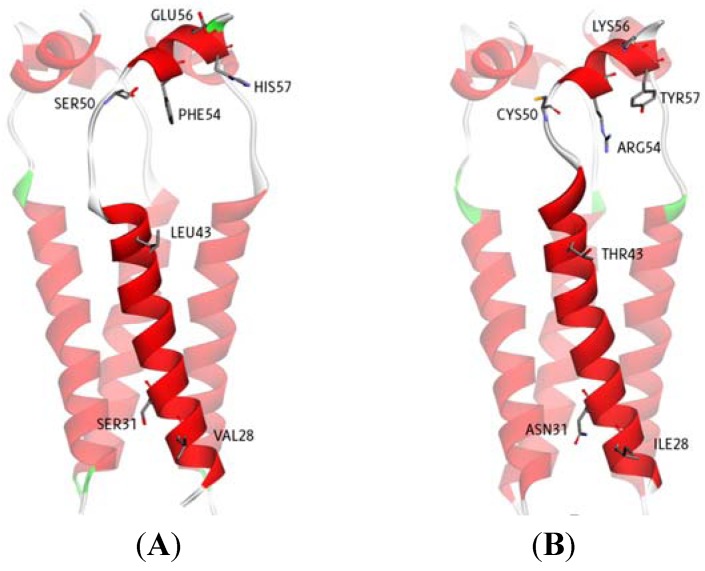
3D Structures of H3N2 M2 channel protein (**A**) and 2009-H1N1 M2 channel protein (**B**) respectively.

The template for the M2 model construction was the high-resolution NMR structure of M2 channel protein (PDB code of 2RLF) [14]. In the M2 model construction of the 2009-H1N1 virus, the M2 protein sequence was taken from Genbank (accession number GQ385303), isolated in July 2009 from an H1N1 virus strain in Japan [10,15] (*cf.* Figure 5). Most synthetic inhibitors of M2 channel proteins, amantadine scaffolds based on adamantane derivatives were selected from published work by Gayday *et al. *[9], Du *et al. *[10], Eleftheratos *et al. *[18], Papanastasiou *et al. *[21], Tanner *et al. *[22], de Clercq [23], Tataridis *et al. * [24], Stamatiou *et al. *[25], Balannik *et al. *[26] and the others were newly created. These ligands have not been investigated for docking with M2 channel proteins either for the inside or outside positions before. 

The design principle of potential target drug-resistant influenza A on M2 mutants aimed to introduce an additional functional group on the aamantadine scaffold, including adding more amine, hydroxyl, linear, cyclohexane, aromatic and benzene groups, in order to increase the binding affinity of M2 inhibitors. The amino group in amantadine is likely the pharmacophore and is necessary to block hydrogen ion transport. Consequently, the scaffold was based on the adamantyl group and selection of suitable functional groups attaching to the scaffold to form new compounds that will potentially bind well to M2 channel proteins at the key residues in the binding site [27]. Prior to molecular docking simulation, all 200 new potential M2 inhibitors were built using Gaussview 4.1 software [28]. All compounds were optimized geometrically using Discovery Studio 3.0 Visualizer [29].

### 3.2. Virtual Screening with Autodock 3.05

In the molecular docking simulations, two types of M2 channel proteins representing H3N2 and 2009-H1N1 virus strains were used as receptor with two different docking positions inside and outside the channel. AutoDock Tools version 1.5.4 [30] was used to add polar hydrogens, assign Kollman charges and create grid binding boxes. The volume of each grid box was 30 × 30 × 30, with the default 0.375 Å spacing. For each type of M2 channel protein structures, AutoGrid version 3.05 was used to create afﬁnity grids which centered at the active sites. Based on the coexistence of two possible binding positions and two existing M2 channel proteins [20], docking inhibitors into M2 channel will be separated into four categories: (1) docking inside the H3N2 M2 channel protein; (2) docking inside the 2009-H1N1 M2 channel protein; (3) docking outside the H3N2 M2 channel protein; and (4) docking outside the 2009-H1N1 M2 channel protein. The positive control for docking was obtained by re-docking amantadine and rimantadine extracted from the NMR structure [14] to locate the binding site on M2 channel protein before docking for 200 ligands. The binding box inside M2 channel proteins was positioned to encompass all three possible binding sites, namely Ala30, Ser31 and Gly34 and the binding box outside M2 channel proteins was set up to cover two important functional residues: channel gate Trp41 and channel lock Asp44 [15]. The binding affinities were calculated by AutoGrid version 3.05 using the following atom types: aromatic carbon (A), carbon (C), fluorine (F), iodine (I), nitrogen (N), hydrogen bond accepting N (NA), oxygen (O), hydrogen bond accepting O (OA), sulfur (S), hydrogen bond accepting (SA), silicon (Si), hydrogen (H) and electrostatic (e) [30]. The ligand set includes 200 new M2 inhibitors created from previously published studies and the others were newly created by modifying or adding more functional groups to the adamantane scaffold. AutoDockTools 1.5.4 was also used to merge nonpolar hydrogens, add charges and visually set up rotatable bonds for each ligand via AutoTors.

A Lamarckian genetic algorithm [31] was used to perform the docking experiments on AutoDock 3.05. The parameters were optimized as follows: trials of 100 dockings, population size of 50, random starting position and conformation, translation step range of 2.0 Å, rotation step range of 50 degrees, maximum number of generations of 27,000, elitism of 1, mutation rate of 2%, crossover rate of 80%, local search rate of 6%, 100,000 energy evaluations and docked conformations were clustered with the tolerance of 1.0 Å RMSD. Docking results were sorted by the lowest binding energy of the most populated cluster using AutoDockTools version 1.5.4. The top 10 hits from each of group binding inside and outside both H3N2 and 2009-H1N1 M2 channel proteins were selected for further analysis.

### 3.3. Pharmacophore Modeling

The top ten bound compounds were selected based on the lowest free energy of binding for pharmacophore analysis to give important insight into interactions between the top hits among M2 channel-inhibitors and common pharmacophore features in each group binding inside and outside. For this purpose, structure- and ligand-based pharmacophore modeling studies were carried out using the LigandScout 3.01 [32] and *HipHop* module of Discovery Studio 2.5 software [33], respectively. LigandScout generates the structure-based pharmacophore model based on the relevant interactions between the protein-ligand whereas Hip-Hop mainly focused on the possible common features present in the set of inhibitors.

#### 3.3.1. Generation of Structure-Based Pharmacophore Models Using LigandScout 3.01

The top ten compounds binding on the inside and outside the M2 channel proteins with the lowest binding energy were used to generate the structure-based pharmacophore models [34]. The M2 channel-inhibitor observations were verified to compare the interactions between binding inside and outside of M2 channel proteins. The ligand interactions with critical amino acids present in the active site of M2 channel proteins pharmacophore based on best result of virtual screening provide a sufficient input to generate the structure-based. LigandScout was used to study the interactions between the M2 inhibitors and the amino acids in the two binding sites of M2 channel, as well as a tool for automatic construction and visualization of structure based pharmacophore model. LigandScout extracts and interprets ligand-receptor interactions such as hydrogen bond, charge transfer, hydrophobic regions of their macromolecular environment. Chemical features include hydrogen-bond donors and acceptors as directed vectors, positive and negative ionizable areas as well as lipophilic areas represented by spheres. In order to increase selectivity, excluded volume spheres are added to reflect potential steric restrictions. The 3D coordination of the interaction was obtained and resulted in speciﬁc interaction model that are able to map the ligands in their bioactive conformation. As a result, from the top 10 compounds binding at both sides, the most important inside interactions that can hold and stabilize the drug inside the M2 channel proteins were selected and visualised.

#### 3.3.2. Ligand-Based Pharmacophore Modeling Using Discovery Studio 2.5

The identification of important common chemical features from the top binding compounds inside and outside M2 channel proteins should be helpful to discover potent candidates to inhibit both the H3N2 and 2009-H1N1 virus strains. The signiﬁcance of pharmacophore models mostly depends on the quality of the molecule structures used in generation of the pharmacophore conformation [35]. In this work, the training set molecule was selected from two different groups: the top 10 compounds binding inside the M2 channel proteins and the top 10 compounds binding outside the M2 channel proteins. The bond orders of these inhibitors were checked and verified before the generation of a pharmacophore model. The conformational flexibility of selected ligands was accomodated by creating multiple conformers to cover all representatives over a specified energy threshold. The HipHop module of Discovery Studio 2.5 software was employed to build a plausible binding hypothesis and identify a set of chemical features common to the most potent training molecule structures [36]. The general chemical features that considered in this training set were hydrogen bond donors and acceptors (HBDs and HBAs), and aliphatic hydrophobes. Eighteen hypotheses were generated in the outside group and seventeen hypotheses were created in the inside group, and the best hypothesis from each group was selected based on the high number of compounds fitting it as well as the high fit value of the hypothesis.

## 4. Conclusions

In this study the top highly potent anti-inﬂuenza A compounds from among 200 new compounds based on the amantadine scaffold were identified by a combination of different computational methodologies. The results also confirmed that the binding inside position was more favourable and stable than binding outside the M2 channel proteins. The results obtained based on virtual screening revealed that the top 10 compounds binding at both positions inside and outside the channel have higher binding affinity to both H3N2 and 2009-H1N1 M2 channel proteins than amantadine and rimantadine. Detailed pharmacophore analysis for the top hits among the M2 channel inhibitors also revealed the nature of interactions between functional groups of the top binding candidates with the M2 channel proteins. From this study, two compounds, I5 and I9, were predicted to be the most active inhibitors against both inﬂuenza A virus strains H3N2 and 2009-H1N1. However, due to the limitations of the method employed, such findings and additional mutation of M2 channel protein studies would require more accurate calculations to be confirmed. Molecular dynamics simulation should be done in the future to capture the flexible interactions between ligand-M2 channel proteins, together with *in vitro *and *in vivo *studies on the top binding compounds.
